# Significant Impact of the MTHFR Polymorphisms and Haplotypes on Male Infertility Risk

**DOI:** 10.1371/journal.pone.0069180

**Published:** 2013-07-18

**Authors:** Nishi Gupta, Saumya Sarkar, Archana David, Pravin Kumar Gangwar, Richa Gupta, Gita Khanna, Satya Narayan Sankhwar, Anil Khanna, Singh Rajender

**Affiliations:** 1 Division of Endocrinology, Central Drug Research Institute, Lucknow, India; 2 Ajanta Hospitals and IVF Centre Pvt. Ltd., Lucknow, India; 3 Department of Urology, King George Medical University, Lucknow, India; Institut Jacques Monod, France

## Abstract

**Background:**

Methylenetetrahydrofolate reductase (MTHFR) converts 5,10-methylene tetrahydrofolate to 5-methyl tetrahydrofolate and affects the activity of cellular cycles participating in nucleotide synthesis, DNA repair, genome stability, maintenance of methyl pool, and gene regulation. Genetically compromised MTHFR activity has been suggested to affect male fertility. The objective of the present study was to find the impact on infertility risk of c.203G>A, c.1298A>C, and c.1793G>A polymorphisms in the MTHFR gene.

**Methods:**

PCR-RFLP and DNA sequencing were used to genotype the common SNPs in the MTHFR gene in 630 infertile and 250 fertile males. Chi-square test was applied for statistical comparison of genotype data. Linkage disequilibrium between the SNPs and the frequency of common haplotypes were assessed using Haploview software. Biochemical levels of total homocysteine (tHcy) and folic acid were measured. Meta-analysis on c.1298A>C polymorphism was performed using data from ten studies, comprising 2734 cases and 2737 controls.

**Results:**

c.203G>A and c.1298A>C were found to be unrelated to infertility risk. c.1793G>A was protective against infertility (P = 0.0008). c.677C>T and c.1793G>A were in significant LD (D’ = 0.9). Folic acid and tHcy level did not correlate with male infertility. Pooled estimate on c.1298A>C data from all published studies including our data showed no association of this polymorphism with male infertility (Odds ratio = 1.035, P = 0.56), azoospermia (Odds ratio = 0.97, P = 0.74), or oligoasthenoteratozoospermia (Odds ratio = 0.92, p = 0.29). Eight haplotypes with more than 1% frequency were detected, of which CCGA was protective against infertility (p = 0.02), but the significance of the latter was not seen after applying Bonferroni correction.

**Conclusion:**

Among MTHFR polymorphisms, c.203G>A and c.1298A>C do not affect infertility risk and c.1793G>A is protective against infertility. Haplotype analysis suggested that risk factors on the MTHFR locus do not extend too long on the DNA string.

## Introduction

Infertility is the inability of a couple to conceive even after one year of regular, unprotected intercourse. The disorder is multifactorial in nature and affects about 15–20% of the couples trying for pregnancy [1]–[2], of which male factors account for about 40% cases [3]. Etiological factors acting at pre-testicular, testicular, or post-testicular level may alter sperm production and/or function [4]–[5]. Genetic factors, such as Y-chromosomal microdeletions, chromosomal anomalies, and copy number variations (CNVs) in the genes involved in testicular function have been identified to be causative or risk factors for male infertility [6]. Hundreds of genes are known to take part in orchestrating the complex process of spermatogenesis. Folate metabolic pathway plays important roles in cellular physiology by participating in nucleotide synthesis, DNA repair and methylation, and maintenance and stability of the genome. High metabolic activity in testes requires optimal functioning of this pathway. A study on adult mouse showed that the key enzyme (methylenetetrahydrofolate reductase) of the folate pathway is five times more active in testes in comparison to other organs, suggesting its critical role in spermatogenesis [7].

Methylenetetrahydrofolate reductase (MTHFR), which catalyzes conversion of 5, 10- methylene tetrahydrofolate to 5-methyl tetrahydrofolate, is an important enzyme of folate pathway. This pathway maintains the methyl pool required for regulatory functions and conversion of homocysteine (hcy) to methionine [8]–[10]. *MTHFR* deficient mice exhibit global DNA hypomethylation, hyperhomocysteinemia and increased *S*-Adenosyl homocysteine (SAH), and most importantly, compromised spermatogenesis [9]. The importance of MTHFR in male fertility is further emphasized by relatively recent research showing altered sperm DNA methylation in infertile individuals [11] and epigenetic regulation of genes involved in spermatogenesis [12]. Increase in sperm concentration upon folic acid and zinc sulfate supplementation further highlights the importance of this pathway in spermatogenesis [13].

Compromised activity of the MTHFR enzyme results in increased homocysteine levels [14]. Hyperhomocysteinemia has been associated with various pathophysiological conditions, such as atherosclerosis, vascular disorders, neural tube defects, Parkinson disorder, pregnancy complications, polycystic ovarian syndrome (PCOS), and male infertility etc.[10], [15]–[17]. Two common polymorphisms, c.677C>T (rs1801133) and c.1298A>C (rs1801131), result in decreased catalytic activity and increased thermolability of the *MTHFR* enzyme [18]–[19]. c.677C>T results in the substitution of alanine by valine, and the enzyme has only 30% residual activity in homozygous condition and about 70% residual activity in heterozygous condition [18]. c.1298A>C results in substitution of glutamic acid by alanine, reducing the enzymatic activity, but to a lesser extent than c.677C>T [18], [20]. c.203G>A and c.1793G>A substitutions result in replacement of arginine by glutamine at 69 and 594 amino acid positions, respectively.

In a recent study, we analyzed c.677C>T polymorphism in an Indian population and conducted a meta-analysis to conclude significantly increased risk of male infertility in carriers of this substitution [21]. Three other polymorphisms (c.203G>A, c.1298 A>C, and c.1793 G>A) in the *MTHFR* gene have been suggested to be candidates for male infertility [2], [22]–[30]. Out of these polymorphisms, c.1298A>C has been analyzed in few other populations [2], [22]–[30], providing us an opportunity to undertake a pooled data analysis. Therefore, we have designed the present study to; i) find male infertility risk associated with c.203G>A, c.1298A>C, and c.1793G>A polymorphisms in the *MTHFR* gene, ii) undertake meta-analysis on c.1298A>C to have a pooled estimate regarding its impact on infertility risk, and iii) correlate biochemical levels of homocysteine and folic acid with infertility.

## Materials and Methods

### Sample Collection

The Institutional Human Ethics Committee of the Ajanta Hospitals and IVF Centre and that of the King George Medical University (KGMU), Lucknow, approved this study. We recruited 630 infertile men from the Ajanta Hospitals and IVF Centre Pvt. Ltd., Alambagh and the Department of Urology, KGMU, Lucknow, for a case-control study. Informed written consent of each patient was obtained in response to a fully written and verbal explanation of nature of the study. Patients suffering from varicocele, diabetes, mumps, and those showing chromosomal anomalies etc. were excluded from the study. Upon examination of semen quality, patients were categorized according to the WHO 1999 criteria (Table S1) [31]. Following the criteria of a normal semen profile and confirmed paternity, 250 male individuals of comparable age were recruited as controls. All patients and controls belonged to Indo-European ethnicity.

### Genotype Analysis

Genomic DNA was isolated from lymphocytes of the peripheral blood of the patient and control samples using the phenol-chloroform-isoamyl method [32]. Three exonic SNPs, c.203G>A (rs2066472), c.1298A>C (rs1801131), and c.1793G>A (CM056008 NOIATAUM_DMCH), and one intronic (rs3818762) SNP in the *MTHFR* gene were genotyped using PCR-RFLP and direct DNA sequencing methods. The methodology and primers used in the study are detailed in Table 1. Briefly, primers around the polymorphic sites were designed with the help of primer-blast tool of the NCBI. PCR was carried out in a total reaction volume of 10 µl each in thin walled tubes, consisting of 1.0 µl of 10X PCR buffer (NEB), 1.0 µl of 10mM dNTPs (Bangalore Genei), 2.0 pM of each of the forward and reverse primers (sequences mentioned in Table 1), 1.0 unit of *Taq* DNA polymerase enzyme (NEB), and 40 ng of genomic DNA. PCR cycling was carried out using ABI Veriti thermal cycler (Applied Biosystems, USA). PCR amplification conditions consisted of denaturation at 95°C for 10 minutes, followed by 35 cycles of denaturation at 95°C for 30 seconds, annealing at a suitable temperature for 30 seconds (detailed in Table 1), and polymerization at 72°C for 40 seconds, and a final stage of polymerization at 72°C for 7 minutes. PCR products for rs2066472 and rs1801131 SNPs were digested with *Taq* I and *Mbo* II enzymes, respectively. Digested products were electrophoresed on 3% agarose gel. Randomly selected samples were subjected to direct DNA sequencing of the polymorphic region to confirm the accuracy of the results. The amplified products for CM056008 NOIATAUM_DMCH and rs3818762 SNPs were directly sequenced using Big-Dye™ chain termination chemistry on ABI 3730 DNA analyzer (Applied Biosystems, USA) [33]. Multiple alignment and sequence analysis were done using the Auto Assembler Software (Applied Biosystems, USA).

**Table 1:** List of the SNPs analyzed along with PCR primers and techniques used for genotyping.

SNP (rs ID)	Amino acidChange	Techniqueadopted	Forward Primer/Reverse Primer (5′-3′)	Annealingtemperature	FragmentSize (bp)
203 G>A (rs2066472)	R68Q	PCR-RFLP(*Taq* I)	CCCTGCTTGGAGGGCAGTGC GCAGATCAGATGACCCACTCTGCCT	67°C	351
1298 A>C (rs1801131)	E429A	PCR-RFLP(*Mbo II*)	CTGCCCTCTGTCAGGAGTGTGC3’/CCCTTCTCCCTTTGCCATGTCCA3’	65°C	368
1793 G>A (CM056008NOIATAUM_DMCH)	R594Q	Sequencing	GTGATACTGGCAGTGGGCCTTGT CTCTCGCATTCTGGGTGGGC	61°C	332
Intronic (rs3818762)	–	Sequencing	GTGATACTGGCAGTGGGCCTTGT CTCTCGCATTCTGGGTGGGC	61°C	332

### Statistical Analysis

Genotype data of control samples for all polymorphisms were analyzed for fitness in the Hardy Weinberg Equilibrium using the online calculator available at http://ihg.gsf.de/cgi-bin/hw/hwa1.pl. Chi square test was used to compare allele and genotype data between cases and controls using Vassar Stats Online Calculator (http://faculty.vassar.edu/lowry/VassarStats.html), adopting dominant, recessive, co-dominant, and additive models. Fisher exact test was applied wherever Chi square test was not applicable. In addition to comparison of all patients with controls, genotype and allele distribution were compared between case groups based on sperm motility (motility <30% and > = 30%) and sperm count (count <20million/ml, > = 20 million/ml and <100 million/ml, > = 100million/ml). A P-value of less than 0.05 was considered to be statistically significant. The significance in case of multiple comparisons was assessed against a reference P value obtained after applying Bonferroni correction.

### Linkage Disequilibrium (LD) and Haplotype Analysis

LD was calculated and plotted using the Haploview software (Version 4.2) developed at ‘The Broad Institute’ (http://www.broadinstitute.org) [34]. For calculation of LD and haplotype frequency, we have included data for c.677C>T SNP from our previous study [21]. Haplotype frequencies were calculated using the same software in order to find if certain allelic combinations of these SNPs affected the risk of infertility.

### Folic Acid and Homocysteine Estimation

Blood samples were collected in serum separator tubes and allowed to clot for collection of serum. Homocysteine was measured in the serum samples using an enzymatic test based kit provided by Globe Diagnostic (Italy). Folic acid level was measured using chemiluminescence based kit from Siemens diagnostic (Germany) on Immulite 1000 reader.

### Meta-analysis

Data on c.1298A>C polymorphism was available for a few other populations as well. Therefore, we have pooled data on c.1298A>C from our study and other published studies, comprising a total of 2734 cases and 2737 controls in order to perform meta-analysis.

- *Identification of relevant studies*
  To identify the case-control studies that analyzed MTHFR c.1298A>C polymorphism in male infertility, we conducted a systematic literature search through “ Pubmed”, “Google Scholar”, and “Scirus” databases using the keywords “MTHFR and male infertility”, “MTHFR 1298A>C and male infertility”, and “folate metabolism and male infertility”. The search was limited to the articles in English language and till September 2012 as the publication date. Retrieved studies were screened to meet the inclusion criteria that: (i) each trial was an independent case-control study, (ii) the purpose of all the studies and statistical methods were similar, (iii) SNP typing was done at high resolution level, and (iv) inclusion of the patients was done according to standard diagnosis parameters. Studies not providing enough information (genotype data), and those not well described were considered for exclusion. The citations of the articles included were screened carefully to identify maximum numbers of relevant studies.
- *Data extraction*
  The articles satisfying the inclusion criteria were read carefully to extract details regarding first author, year of publication, ethnicity, total number of cases and controls, genotype and allele frequency, and the type of infertility phenotype. Data extraction was performed by NG and independently confirmed by SS.
- *Statistical analysis*
  Meta-analysis was performed using the Comprehensive Meta-Analysis (CMA) software (Version 2), which allows data entry in different formats. Odds ratio was chosen as a measure of the effect size. Heterogeneity was assessed using Chi square based ‘Q’ test, considering P-values <0.10 to be statistically significant [35]. Quantitative assessment of heterogeneity was done by comparing the magnitude of I^2^ value with the classification given by Higgins and Thompson; viz. 25%, 50% and 75%, which correspond to low, medium, and high heterogeneity, respectively [36]. In the absence of heterogeneity, fixed-effect model using the Mantel–Haenszel method was used for pooled estimate; otherwise, a random-effect model using the Der Simonian and Laird method was applied [35]–[36]. However, these two models provide similar results in the absence of heterogeneity between studies. High resolution plot (forest plot) was generated to estimate pooled odds ratio and P-value. P-value <0.05 was considered to be statistically significant. Sensitivity analysis was conducted to validate the assumptions and the decisions made, and for assessing robustness of the analysis method used. Subgroup analysis according to infertility phenotype (azoospermia and OAT) was carried out to estimate phenotype specific effect.
  Publication bias was investigated using the funnel plot of precision by log odds ratio method. Asymmetry of the funnel plot was assessed by Egger’s regression intercept test. Egger’s test estimates bias using precision (inverse of the standard error) to predict standardized effect (effect size divided by the standard error), and significance of the intercept was determined using ‘t’ test, considering P value of <0.05 to be significant.

## Results

### Case-Control Study

We have analyzed four polymorphisms in the MTHFR gene in 630 infertile men and 200 fertile controls by PCR-RFLP and direct DNA sequencing methods. MTHFR genotypes distribution among control samples fitted well in the Hardy-Weinberg equilibrium for c.1298A>C (0.31), c.1793G>A (1.00), and intronic (0.65) polymorphisms, but not for c.203G>A polymorphism. The frequency of allele ‘A’ or genotype ‘GA+AA’ at c.203G>A locus were 0% and less than 1%, respectively, in both fertile and infertile individuals (Table 2), and genotype distribution between the two groups was not significantly different (P = 0.45) (Table 2). The frequency of alleles (‘C’ and ‘G’) and genotypes (‘CC’, ‘CG’ and ‘GG’) for intronic polymorphism were not significantly different between cases and controls. Analysis using 3×2 and 2×2 contingency tables showed no effect of c.1298 A>C polymorphism on infertility risk (Table 2). The frequency of allele ‘A’ at c.1793 G>A locus was significantly lesser in cases (12.33%) in comparison to controls (20.23%) (Table 2), suggesting ‘A’ allele to be a protective allele. Similarly, significant difference in the distribution of genotypes between cases and controls was seen (Table 2), such that individuals with ‘GG’ genotype were at increased risk of infertility. The differences remained statistically significant even after Bonferroni correction (P<0.0083) (Table 2).

**Table 2:** Genotype and allele distribution of MTHFR SNPs in cases and controls.

SNP	Genotype	ControlN (%)	CasesN (%)	11vs12	11vs22	11vs(12+22)	22vs12	22vs(12+11)	11vs12vs22	1vs2
**203G>A** **(rs2066472)**	GG(11)	201(100)	618 (99.19)	P = 0.58	P = 1.00	P = 0.34	P = 1.0	P = 1.0	P = 0.45	–
	GA(12)	0 (0)	3 (0.48)							
	AA(22)	0 (0)	2 (0.32)							
	G(1)	402(100)	1239(99.44)							
	A(2)	0 (0)	7(0.56)							
**1298A>C** **(rs1801131)**	AA(11)	27(19.85)	165(27.0)	OR = 0.71(CI = 0.44–1.14)P = 0.16	0.59(0.34–1.02)0.06	0.67(0.42–1.06)0.08	1.20(0.76–1.89)0.43	1.33(0.87–2.05)0.18	0.16	0.78(0.60–1.02)0.07
	AC(12)	74(54.41)	320(52.37)							
	CC(22)	35(25.73)	126(20.62)							
	A(1)	128(47.05)	650(53.19)							
	C(2)	144(52.95)	572(46.81)							
**1793G>A** **(CM056008** **NOIATAUM_DMCH)**	GG(11)	110(63.58)	293(78.55)	0.46(0.30–0.69)0.000	0.64(0.25–1.68)0.36	0.48(0.32–0.71)0.000	0.71(0.26–1.92)0.50	1.27(0.49–3.28)0.62	0.0008	0.55(0.39–0.78)0.000
	GA(12)	56(32.37)	68(18.23)							
	AA(22)	7(4.05)	12(3.22)							
	G(1)	276(79.77)	654(87.67)							
	A(2)	70(20.23)	92(12.33)							
**Intronic** **(rs3818762)**	CC(11)	52(30.41)	137(35.86)	0.78(0.52–1.18)0.25	0.78(0.47–1.29)0.33	0.78(0.53–1.15)0.21	1.00(0.63–1.61)1.0	1.11(0.71–1.73)0.64	0.45	0.86(0.67–1.12)0.26
	CG(12)	82(47.95)	169(44.24)							
	GG(22)	37(21.64)	76(19.90)							
	C(1)	186(54.39)	443(57.98)							
	G(2)	156(45.61)	321(42.02)							

To find a correlation of each SNP with semen parameters, we cross-classified genotype data with reference to sperm motility and count (Tables 3 and 4). Genotype distribution did not differ significantly between groups with low (<30%) and high (> = 30%) sperm motility (Table 3), or between groups with low (<20 million/ml), average (> = 20 million/ml and <100 million/ml), and high (> = 100 million/ml) sperm counts (Table 4). However, allele ‘A’ at c.203G>A locus correlated with a higher sperm count (P = 0.007).

**Table 3:** Genotype and allele distribution of MTHFR SNPs in relation to sperm motility.

SNP/rsID	Genotype	Motility<30%	Motility>30%	11vs12	11vs22	11vs(12+22)	22vs12	22vs(12+11)	11vs12vs22	1vs2
**203G>A** **(rs2066472)**	GG(11)	186(100)	277(98.93)	1.0	0.52	0.28	1.0	0.52	0.37	0.16
	GA(12)	0(0)	1(0.36)							
	AA(22)	0(0)	2(0.71)							
	G(1)	372(100)	555(99.11)							
	A(2)	0(0)	5(0.89)							
**1298A>C** **(rs1801131)**	AA(11)	29(25.66)	77(25.67)	OR = 1.01(CI = 0.60–1.7)P: 1.0	0.98(0.52–1.83)0.92	1.00(0.61–1.64)1.0	1.04(0.60–1.79)0.89	1.03(0.61–1.74)0.92	0.99	0.99(0.73–134)1.0
	AC(12)	59(52.21)	155(51.67)							
	CC(22)	25(22.12)	68(22.67)							
	A (1)	117(51.77)	309(51.5)							
	C(2)	109(48.23)	291(48.5)							
**1793G>A** **(CM056008** **NOIATAUM_DMCH)**	GG(11)	50(67.57)	126(78.75)	1.87(0.96–3.63)0.06	1.44(0.40–5.13)0.73	1.78(0.69–3.30)0.07	1.3(0.33–5.04)0.75	0.80(0.23–2.82)0.75	0.17	1.59(0.94–2.69)0.08
	GA(12)	20(27.03)	27(16.88)							
	AA(22)	4(5.41)	7(4.38)							
	G(1)	120(81.08)	279(87.19)							
	A(2)	28(18.92)	41(12.81)							
**Intronic** **(rs3818762)**	CC(11)	24(30.38)	61(37.42)	1.50(0.80–2.81)0.20	1.18(0.57–2.42)0.65	1.37(0.77–2.44)0.28	1.27(0.64–2.52)0.49	1.06(0.57–1.98)0.86	0.44	1.13(0.77–1.65)0.54
	CG(12)	36(49.37)	61(37.42)							
	GG(22)	19(24.05)	41(25.15)							
	C(1)	84(53.16)	183(56.13)							
	G(2)	74(46.84)	143(43.87)							

**Table 4:** Genotype and allele distribution of MTHFR SNPs in relation to sperm count.

SNP/rsID	Genotype	Count <20 M/ml	Count >20 M/ml and <100 M/ml	Count>100 M/ml	11vs12	11vs22	11vs(12+22)	22vs12	22vs(12+11)	11vs12vs22	1vs2
**203G>A (rs2066472)**	GG(11)	100(100)	273(100)	185(98.40)	P = 0.37	0.14	0.05	1.0	0.14	0.20	0.007*
	GA(12)	0(0)	0(0)	1(0.53)							
	AA(22)	0(0)	0(0)	2(1.06)							
	G(1)	200(100)	546(100)	371(98.67)							
	A(2)	0(0)	0(0)	5(1.33)							
**1298A>C (rs1801131)**	AA(11)	15(18.07)	44(29.33)	54(25.47)	0.15	0.4	0.17	0.66	0.73	0.35	0.41
	AC(12)	50(60.24)	75(50.0)	107(50.47)							
	CC(22)	18(21.69)	31(20.67)	51(24.06)							
	A(1)	80(48.19)	163(54.33)	215(50.71)							
	C(2)	86(51.81)	137(45.67)	209(49.29)							
**1793G>A (CM056008 NOIATAUM_DMCH)**	GG(11)	29(70.73)	56(74.67)	103(78.63)	0.46	0.51	0.55	0.43	0.50	0.57	0.53
	GA(12)	9(21.95)	17(22.67)	21(16.03)							
	AA(22)	3(7.32)	2(2.67)	7(5.34)							
	G(1)	67(81.71)	129(86.0)	227(86.64)							
	A(2)	15(18.29)	21(14.0)	35(13.36)							
**Intronic (rs3818762)**	CC(11)	13(30.95)	31(38.75)	46(34.07)	0.89	0.45	0.75	0.67	0.50	0.80	0.4
	CG(12)	17(40.48)	33(41.25)	54(40.0)							
	GG(22)	12(28.57)	16(20.0)	35(25.93)							
	C(1)	43(51.19)	95(59.38)	146(54.07)							
	G(2)	41(48.81)	65(40.63)	124(45.93)							

c.203G>A had a minor allele frequency (MAF) of <1%; therefore, only three SNPs in the coding region and one intronic SNP were included in LD and haplotype analysis. The data for c.677C>T site from our previous study [21] were included in LD calculation. c.1298A>C and c.1793G>A polymorphisms were not in significant LD (D′ = 0.453, confidence bound = 0.28–0.6, LOD = 4.4, r^2^ = 0.04). c.677C>T and c.1298A>C polymorphisms (D′ = 0.586, confidence bound = 0.41–0.72, LOD = 6.32, r^2^ = 0.056) showed intermediate concentration of LD. However, c.677C>T and c.1793G>A polymorphisms were in significant LD (D′ = 0.903, confidence bound = 0.58–0.98, LOD = 4.04, r^2^ = 0.024). c.1793G>A and intronic polymorphisms were also in significant LD (D′ = 0.912, confidence bound = 0.82–0.96, LOD = 32.35, r^2^ = 0.196) (Figure 1). Eight haplotypes with a frequency of more than 1% were detected (CACG = 30.7%, CCGG = 23.0%, CCCG = 11.7%, TACG = 11.1%, CCGA = 10.0%, CAGG = 5.0%, CAGA = 4.4% and TCCG = 2.1%) (Table 5). The distribution of all haplotypes, except CCGA, was not significantly different between cases and controls (Table 5). However, after applying Bonferroni correction, none of the haplotypes showed a significant association with infertility.

**Figure 1:**
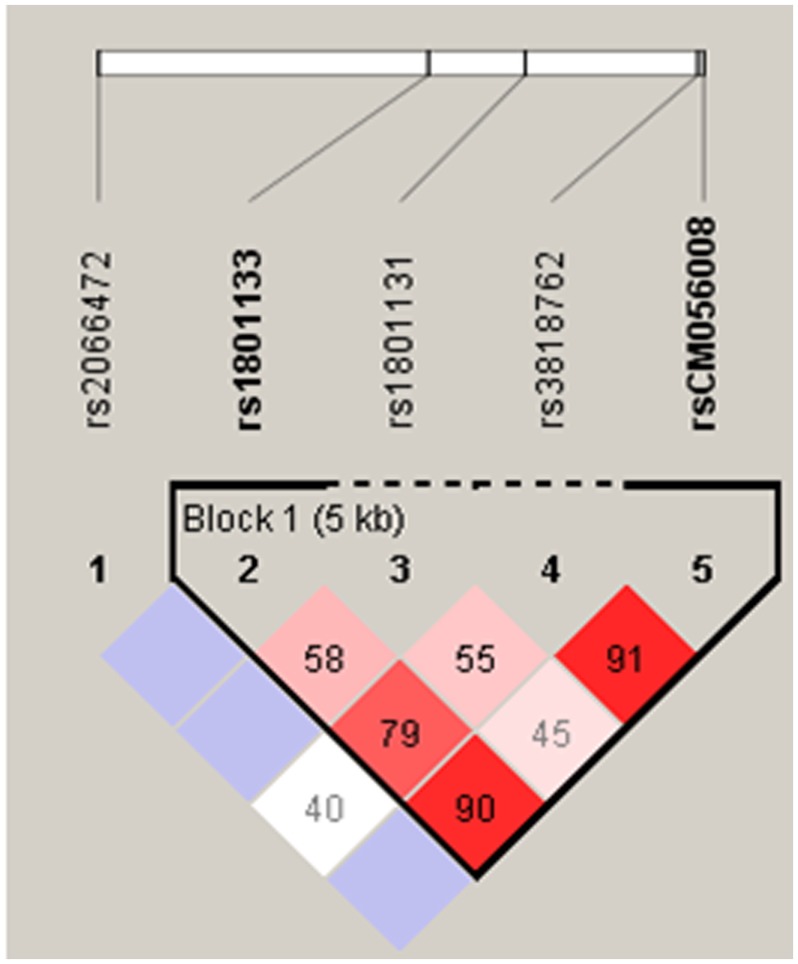
Linkage disequilibrium plot. The number in each cell represents the LD parameter D′ (×100). Each cell is color graduated related to the strength of LD between the two markers. The rs numbers are SNP IDs taken from the Ensembl database. rs1801133, CM056008 NOIATAUM_DMCH, CM056008 NOIATAUM_DMCH, and rs3818762 are in strong LD.

**Table 5:** Common MTHFR haplotypes in relation to male infertility.

Haplotype	Frequency (overall)	Frequency (case, control)	Chi square	P value
CACG	0.307	0.311, 0.299	0.193	0.6602
CCGG	0.23	0.237, 0.210	1.231	0.2672
CCCG	0.117	0.115, 0.121	0.103	0.7482
TACG	0.111	0.120, 0.085	3.642	0.0563
CCGA	0.1	0.089, 0.130	5.371	0.0205*
CAGG	0.05	0.049, 0.052	0.051	0.8213
CAGA	0.044	0.038, 0.060	3.462	0.0628
TCCG	0.021	0.020, 0.025	0.329	0.5664

### Biochemical Assays

We measured homocysteine and folic acid levels in a subset of infertile and fertile individuals. Folic acid level ranged from 6.2 to 17.8µg/L in infertile and from 6.8 to 17.1 µg/L in fertile men. Average folic acid level in infertile and fertile groups was 12.05µg/L and 11.97µg/L, respectively, with no significant difference between the two groups (Table 6). tHcy level ranged from 6.26 to 13.35µmol/L in infertile and from 7.39 to 24.76µmol/L in fertile men. Average tHcy level in infertile individuals (9.30µmol/L) was lower than fertile individuals (15.23µmol/L), with a statistically significant difference (P<0.0001) (Table 6).

**Table 6:** Folic acid and homocysteine concentration in cases and controls.

Biochemicalparameter	Statistical parameter	Cases	Control
**Folic Acid** **(µg/L)**	Mean (M)	12.05	11.97
	Standard Deviation (SD)	4.01	3.69
	Variance	3.91	3.58
	t/df/p-value	+0.07/37/0.95	
**Homocysteine** **(µmol/L)**	Mean (M)	9.30	15.23
	Standard Deviation (SD)	2.03	5.14
	Variance	1.98	4.99
	t/df/p-value	−4.87/37/<0.0001	

### Meta-analysis

**Figure 2:**
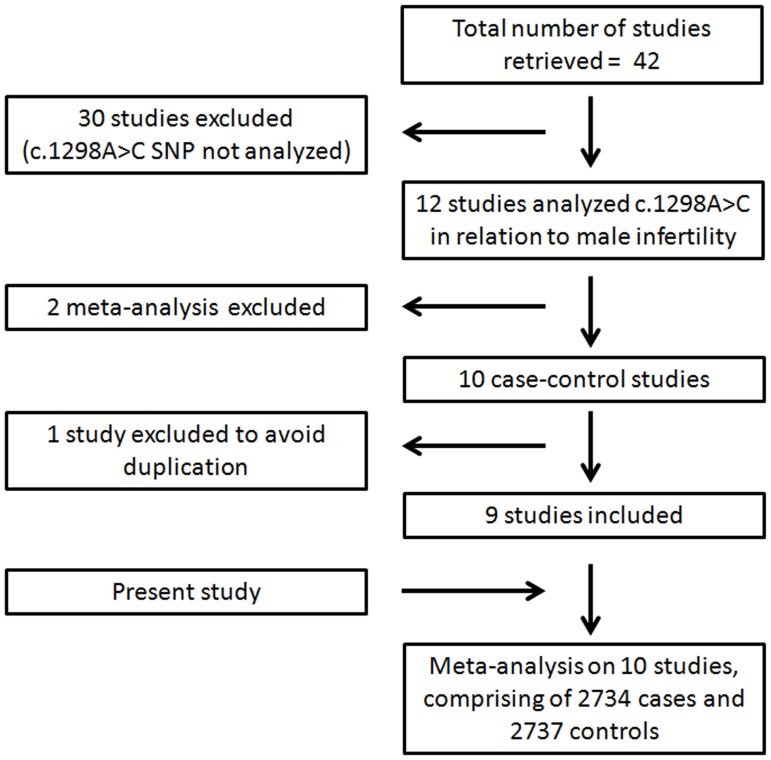
Flow diagram showing inclusion and exclusion of the studies for meta-analysis. Studies retrieved upon literature search were subjected to inclusion/exclusion criteria as detailed in the methods section.

**Figure 3:**
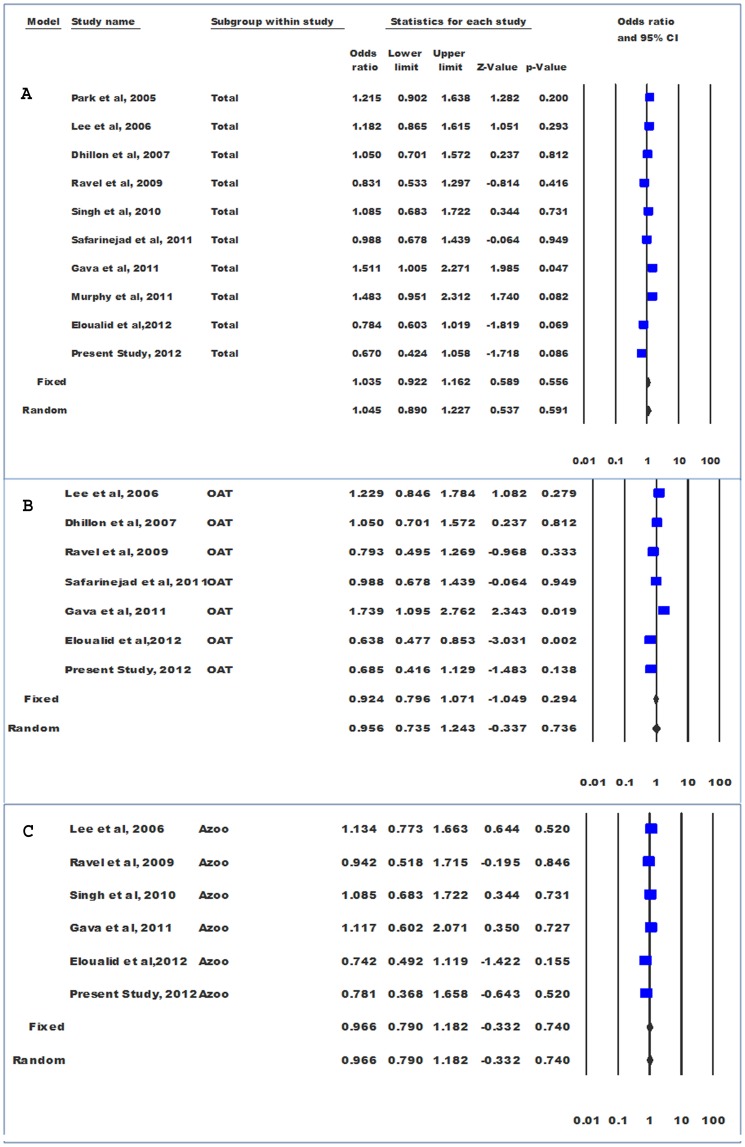
Forest plots. Meta-analysis on c.1298A>C SNP in infertility (A), oligoasthenoteratozoospermia (B), and azoospermia (C).

**Figure 4:**
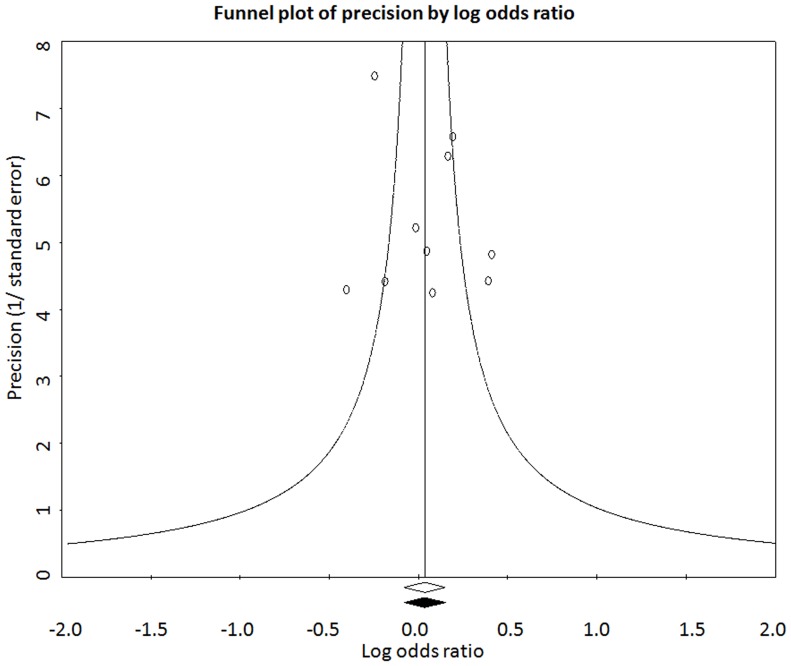
Funnel plot. Plot of precision by log odds ratio using fixed effect model for observed and imputed sets of studies.

- *Literature search*
  Using strictly defined criteria, we could retrieve forty two studies. Few studies had used MTHFR in connection with measurement of vitamin B12, folic acid, and homocysteine. A large number of studies had not analyzed c.1298A>C polymorphism. Few others were either review articles or were conducted on animal models. We found only ten case-control studies looking for correlation of c.1298A>C polymorphism with male infertility [2], [22]–[30]. Two of these studies came from the same research group [2], [29], of which only one [2] was included to avoid duplication. Thus, along with our study, meta-analysis was performed on ten studies, comprising a total of 2734 cases and 2737 controls (Figure 2). Data extracted from each study was tabulated in the supplementary table (Table S2).
- *Quantitative data synthesis*
  Genotype comparison was made adopting dominant model (‘AC+CC’ vs ‘AA’). Significant level of between studies heterogeneity was observed (P_heterogeneity_ = 0.058, Q = 16.47, df(Q) = 9, I^2^ = 45.35, var = 0.029, τ^2^ = 0.0310 SE = 0.031, τ = 0.172). Random effects model was used to pool the data; however, the results of both the models were similar (Fixed OR = 1.035 and Random OR = 1.045). Pooled odds ratio did not show significant association of “AC+CC” genotype with male infertility (OR = 1.05; 95%CI = 0.89–1.23; P = 0.59) (Figure 3).
  Genotype data were stratified according to infertility phenotype (OAT and azoospermia) in order to calculate group-wise effect sizes. The data for azoospermic individuals were homogenous (P_heterogeneity_ = 0.697; I^2^ = 0.00), and pooled odds ratio did not show significant association of “AC+CC” genotype with azoospermia (OR = 0.966; 95%CI = 0.790–1.18; p = 0.740; z = −.0.332). However, in case of OAT, the data were heterogeneous (I^2^ = 66.57, P_heterogeneity_ = 0.006) and no significant association of “AC+CC” genotype with OAT, adopting either fixed (OR = 0.92; 95%CI = 0. 80–1.07; p = 0.29; z = −1.05) or random (OR = 0.96; 95%CI = 0. 74–1.24; p = 0.74; z = −0.34) effects model, was observed (Figure 3).
  Since the control data for three studies [26], [28], [30] were not in the Hardy-Weinberg equilibrium, meta-analysis was also performed after exclusion of these studies. Their exclusion rendered the data more homogenous (P_heterogeneity_ = 0.16, Q = 9.22, df(Q) = 6, I^2^ = 34.09, var = 0.001, τ^2^ = 0.02, SE = 0.033, τ = 0.14); however, there was no change in the conclusion (OR = 1.08; 95%CI = 0. 94–1.24; p = 0.30; z = 1.04).
  Funnel plot and Egger’s test were used to quantify publication bias. The distribution of the studies on the funnel plot did not reveal any evidence of asymmetry, suggesting the absence of bias in quantitative assessment of the pooled data (Figure 4). The absence of bias was confirmed by Egger’s regression intercept test (t = 0.45; Intercept = 1.0; SE = 2.24 and p = 0.67).
  Similarly, meta-analysis comparing allele distribution did not show an association of c.1298A>C polymorphism with male infertility (P = 0.495), OAT (P = 0.831) and azoospermia (P = 0.864).

## Discussion

The impact of MTHFR gene SNPs on infertility risk has been studied in various ethnic groups [2], [13], [22]–[25], [27], [29], [37]–[43]. Most commonly studied MTHFR SNP, c.677C>T, has been established as a risk factor for male infertility in some populations [21–23, 37 and 39–41], but not in others [13], [24]–[25], [38], [42]–[43]. c.677C>T has been suggested to infertility risk by two meta-analyses [3 and 21], but denied doing so by a recent meta-analysis [44]. A population specific meta-analysis may help to resolve the controversy regarding association of this polymorphism with male infertility. In contrast to previous findings, we have found that the presence of ‘A’ allele at 1793 locus is protective [27], [29]. Also, individuals with 1793-G allele showed significantly higher serum tHcy and lower folate levels, suggesting 1793G to favor adverse outcome [27]. The present investigation did not find any impact of c.203G>A substitution on infertility risk in the study population. This is also supported by a previous study conducted on a French population [25]. However, the interpretation regarding c.203G>A should be taken with a caution as the control data were not in the Hardy–Weinberg equilibrium.

A to C transversion at the 1298 locus does not affect infertility risk in Indian men. The results of our study are in concordance with a previous study conducted on Indian population [24]. Another Indian study reported a marginal difference in the frequency of ‘CC’ and ‘AA’ genotypes between cases and controls (P<0.05) [26]. Similarly, the frequencies of ‘AC’ and ‘CC’ did not differ significantly between fertile and infertile Korean men [22]–[23]. Lack of association between c.1298A>C and male infertility was seen in French and Moroccan populations as well [25], [30]. Nevertheless, in Moroccan population, the frequency of ‘CC’ was higher in infertile individuals than fertile, and a statistically significant level of difference was reported in comparison between severe oligozoospermic cases and controls (P = 0.014) [30]. Interestingly, contrasting outcomes have been reported in Brazilian men in two studies by the same group [2], [29]. Ravel et al have suggested that the observed association could be due to population stratification instead of a causal link with the phenotype, as the frequency of allele ‘C’ at c.1298A>C locus differs across populations; especially in Chinese and Caucasians [25]. Marginal association in some of the sub-groups suggests the association to be likely due to population stratification instead of a true causal relationship.

To dissect the nature of association between c.1298A>C polymorphism and male infertility, we performed a meta-analysis on data pooled from all published studies. SNP data on 2734 cases and 2737 controls from 10 cases-controls studies were included in the pooled estimate. In overall analysis, 1298 genotype (AC+CC) did not show any association with infertility. Similarly, a stratified analysis on the basis of infertility phenotype did not reveal any significant association between this SNP and azoospermia or OAT. We could not conduct analysis on the basis of ethnicity due to lack of many studies from a particular ethnic group. Exclusion of the studies not following the Hardy-Weinberg equilibrium [26], [28], [30] did not change the conclusion. Another recent meta-analysis has published similar conclusion [45]. Nevertheless, a previous meta-analysis suggested this SNP to affect the risk of azoospermia with marginal significance [46]. We found this SNP to be unrelated to infertility, which is also supported by pooled estimate on infertility and azoopermia/OAT subgroups. Out of four SNPs, c.677C>T and c.1793G>A were in significant LD, and we found both to affect infertility risk. Out of four major haplotypes (CACG, CCGG, CCCG and TACG) that constitute almost 80% of all haplotypic diversity, none was found to affect infertility risk. Rather CCGA haplotype was significantly more frequent in controls than cases (p = 0.02), suggesting its protective impact. Therefore, it is apparent that the *MTHFR* risk alleles are isolated and do not stretch too long on the DNA string.

Folic acid supplementation is known to reduce Hcy level and the incidence of birth defects in women, but evidently it also serves an important complementary function in men [47]–[48]. A significant increase in sperm count and motility was observed after folate supplementation in two different controlled trials [49]–[50]. It has been reported that individuals with low sperm count have low folate level in seminal plasma and show sperm DNA damage [51]. However, we have observed no significant difference in folate level between fertile and infertile men. It has been proposed that increased tHCy level may correlate with infertility [24]; however, we observed significantly higher tHCy levels in fertile men. The results are in concordance with previous findings showing no association between folate, cobalamin, tHCy and male infertility [28 and 52].

In conclusion, the presence of ‘T’ at locus 677 increases the risk of infertility. On the other hand, ‘A’ allele at locus 1793 is protective against infertility in Indian men, but c.203G>A and c.1298A>C do not correlate with fertility status. The exact mechanism underlying the impact of folate pathway on fertility is not yet clear; however, some possible mechanisms have been put forward. Reduced MTHFR activity due to inadequate intake of folate and vitamin B12 or due to genetic alterations may lead to hyperhomocysteinemia, which might cause auto-oxidation resulting in oxidative stress [53]. The latter is well known to cause damage to sperm DNA and membrane [54]. We did not find increased homocysteine level in the infertile individuals, but the above mechanism may partially explain MTHFR associated infertility risk. Since several genes involved in spermatogenesis are regulated by DNA methylation [26], alteration in methylation pattern could result in global hypomethylation of the genome [13], providing an alternate explanation for the relation between MTHFR gene and infertility. The induction of hypo-methylation by 5-aza deoxycytidine inhibited the differentiation of spermatogonia into spermatocytes in a murine model [55], lending further support to the above hypothesis. Friso and Choi suggested that interaction between nutritional status and genetic polymorphisms could modulate gene expression through DNA methylation, especially when genetic polymorphisms limit the supply of methyl groups [56].

## Supporting Information

Table S1 **Categorization of infertile patients: Infertile patients were categorized into groups following the WHO (1999) criteria.**
(DOCX)

Table S2 **Meta-analysis: Summary of the studies included in the meta-analysis on c.1298A>C polymorphism.**
(DOCX)
