# Supplementary material for: Significant Impact of the MTHFR Polymorphisms and Haplotypes on Male Infertility Risk
Source: PLoS One. 2013 Jul 18;8(7):e69180. doi: 10.1371/journal.pone.0069180 (PMC3715460; doi:10.1371/journal.pone.0069180)
Supplement: Table S1 — Categorization of infertile patients: Infertile patients were categorized into groups following the WHO (1999) criteria. (DOCX) [file pone.0069180.s001.docx]

**Table S1: Categorization of infertile patients according to the WHO (1999) criteria**

| **Infertility phenotype / controls** | **Semen parameters** | **Number of individuals** |
| --- | --- | --- |
| Azoospermia | No sperm in the ejaculate | 54 |
| Asthenozoospermia | Sperm count >= 20 million/ml, motility < 50% and normal morphology >30% | 226 |
| Normozoospermia | Sperm count >= 20 million/ml, motility >= 50% and normal morphology > 30% | 149 |
| Oligozoospermia | Sperm count < 20 million/ml, motility >=50% and normal morphology >30% | 48 |
| Oligoasthenoteratozoospermia | Sperm count < 20 million/ml, motility < 50% and normal morphology < 30% | 20 |
| Uncategorized | One or the other parameter missed | 133 |
| Fertile controls | Sperm count >= 20 million/ml, motility >= 50% and normal morphology > 30% | 250 |
