# Supplementary material for: Significant Impact of the MTHFR Polymorphisms and Haplotypes on Male Infertility Risk
Source: PLoS One. 2013 Jul 18;8(7):e69180. doi: 10.1371/journal.pone.0069180 (PMC3715460; doi:10.1371/journal.pone.0069180)
Supplement: Table S2 — Meta-analysis: Summary of the studies included in the meta-analysis on c.1298A>C polymorphism. (DOCX) [file pone.0069180.s002.docx]

**Table S2: Summary of all the studies included in the meta-analysis on c.1298A>C polymorphism**

| **Study** | **Population** | **Group** | **Cases** | | | | | | | **Control** | | | | | | |
| --- | --- | --- | --- | --- | --- | --- | --- | --- | --- | --- | --- | --- | --- | --- | --- | --- |
|  |  |  | **Total** | **AA** | **AC** | **CC** | **AC+CC** | **A** | **C** | **Total** | **AA** | **AC** | **CC** | **AC+CC** | **A** | **C** |
| Park et al, 2005 | Korean | **Total** | 373 | 237 | 118 | 18 | 136 | 592 | 154 | 396 | 269 | 111 | 16 | 127 | 649 | 143 |
| Lee et al, 2006 | Korean | **Azoo** | 174 | 109 | 57 | 8 | 65 | 275 | 73 | 325 | 213 | 98 | 14 | 112 | 524 | 126 |
|  |  | **OAT** | 186 | 113 | 63 | 10 | 73 | 289 | 83 | 325 | 213 | 98 | 14 | 112 | 524 | 126 |
|  |  | **Total** | 360 | 222 | 120 | 18 | 138 | 564 | 156 | 325 | 213 | 98 | 14 | 112 | 524 | 126 |
| Dhillon et al, 2007 | Indian | **Total (OAT)** | 179 | 90 | 80 | 9 | 89 | 260 | 98 | 200 | 103 | 84 | 13 | 97 | 290 | 110 |
| Ravel et al, 2009 | French | **Azoo** | 69 | 34 | 28 | 7 | 35 | 96 | 42 | 113 | 54 | 46 | 13 | 59 | 154 | 72 |
|  |  | **OAT** | 181 | 97 | 66 | 18 | 84 | 260 | 102 | 113 | 54 | 46 | 13 | 59 | 154 | 72 |
|  |  | **Total** | 250 | 131 | 94 | 25 | 119 | 356 | 144 | 113 | 54 | 46 | 13 | 59 | 154 | 72 |
| Singh et al, 2010 | Indian | **Total (Azoo)** | 151 | 66 | 76 | 9 | 85 | 208 | 94 | 140 | 64 | 74 | 2 | 76 | 202 | 78 |
| Safarinejad et al, 2011 | Iranian | **Total (OAT)** | 164 | 75 | 70 | 19 | 89 | 220 | 108 | 328 | 149 | 141 | 38 | 179 | 439 | 217 |
| Gava et al, 2011a | Brazillian | **Azoo** | 49 | 26 | 14 | 9 | 23 | 66 | 32 | 233 | 130 | 89 | 14 | 103 | 349 | 117 |
|  |  | **OAT** | 107 | 45 | 48 | 14 | 62 | 138 | 76 | 233 | 130 | 89 | 14 | 103 | 349 | 117 |
|  |  | **Total** | 156 | 71 | 62 | 23 | 85 | 204 | 108 | 233 | 130 | 89 | 14 | 103 | 349 | 117 |
| Murphy et al, 2011 |  | **Total** | \|  \| 58 \| 77 \| 11 \| 88 \| 193 \| 99 \| 176 \| 87 \| 62 \| 27 \| 89 \| 236 \| 116 \| \| --- \| --- \| --- \| --- \| --- \| --- \| --- \| --- \| --- \| --- \| --- \| --- \| --- \| --- \|   146 | 58 | 77 | 11 | 88 | 193 | 99 | 176 | 87 | 62 | 27 | 89 | 236 | 116 |
| Eloualid et al, 2012 | Moroccan | **Azoo** | 110 | 67 | 39 | 4 | 43 | 173 | 47 | 690 | 370 | 303 | 17 | 320 | 1043 | 337 |
|  |  | **OAT** | 234 | 138 | 83 | 13 | 96 | 359 | 109 | 690 | 370 | 303 | 17 | 320 | 1043 | 337 |
|  |  | **Total** | 344 | 205 | 122 | 17 | 139 | 532 | 156 | 690 | 370 | 303 | 17 | 320 | 1043 | 337 |
| Present Study, 2012 | Indian | **Total** | 611 | 165 | 320 | 126 | 446 | 650 | 572 | 138 | 27 | 74 | 35 | 109 | 128 | 144 |
|  |  | **Azoo** | 54 | 13 | 33 | 8 | 41 | 59 | 49 | 138 | 27 | 74 | 35 | 109 | 128 | 144 |
|  |  | **OAT** | 275 | 73 | 141 | 61 | 202 | 287 | 263 | 138 | 27 | 74 | 35 | 109 | 128 | 144 |
|  |  | **Normo** | 149 | 36 | 79 | 34 | 113 | 151 | 147 | 138 | 27 | 74 | 35 | 109 | 128 | 144 |
|  |  | **Unncat** | 133 | 43 | 67 | 23 | 90 | 153 | 113 | 138 | 27 | 74 | 35 | 109 | 128 | 144 |
